# Combined anti-PD-1 and amphotericin B therapy reduces fungal burden and enhances control of murine paracoccidioidomycosis

**DOI:** 10.3389/fcimb.2026.1769296

**Published:** 2026-04-02

**Authors:** Nycolas Willian Preite, Filipe Nogueira Franco, Bianca Vieira dos Santos, Ana Claudia Soares dos Santos, Coral Molist-Homs, Bruno Montanari Borges, Vera Lúcia Garcia Calich, Flavio Vieira Loures

**Affiliations:** 1 Institute of Science and Technology, Federal University of São Paulo, São José dos Campos, Brazil; 2 Department of Biomedical Sciences and Pathobiology, Virginia Tech University, Blacksburg, VA, United States; 3 Department of Immunology, Institute of Biomedical Sciences, University of São Paulo (USP), São Paulo, Brazil

**Keywords:** checkpoint blockade, immunosuppression, immunotherapy, paracoccidioidomycosis, PD-1

## Abstract

Paracoccidioidomycosis (PCM) is a systemic fungal infection caused by *Paracoccidioides* spp. It is endemic to the Americas, with the highest incidence reported in Brazil. Pulmonary involvement occurs in nearly all adult patients. The current standard of care relies on antifungal agents such as amphotericin B (AmB), itraconazole, and fluconazole. However, long-term treatment is often associated with poor patient adherence and sequelae, attributable to drug toxicity, chronic inflammation, and fibrosis, which can impair organ function. In this context, therapies that modulate the host immune response have gained prominence. Antibodies targeting the PD-1 immune checkpoint, a regulatory protein highly expressed on lymphocytes, represent a particularly promising strategy. Previous work from our group demonstrated that anti-PD-1 administration in *P. brasiliensis*-infected mice led to controlled disease, characterized by a reduced fungal burden and improved survival. Notably, this clinical improvement correlated with the preservation of protective Th1/Th17 responses. Based on these findings, this study aimed to evaluate the efficacy of a combined therapy using anti-PD-1 alongside a conventional antifungal. We sought to assess its impact on host immune modulation and fungal clearance. To this end, C57BL/6 mice were inoculated with 1×10^6^ *P. brasiliensis* yeast cells. After six weeks, the mice were treated with anti-PD-1, either alone or in combination with AmB. The disease course was evaluated for two weeks post-treatment through CFU counts, histological analysis, and survival monitoring. The host immune response in the lungs was characterized using ELISA and flow cytometry. Our results demonstrate that the anti-PD-1 + AmB combination led to superior disease control compared to monotherapies. This was evidenced by a significant reduction in fungal load, diminished pulmonary lesion size, and enhanced survival. Furthermore, the combined treatment promoted an increase in the pulmonary effector lymphocyte population while reducing overall cytokine levels, indicating effective pathogen control without excessive inflammation. Collectively, these data indicate that restoring an effective immune response via PD-1 blockade, in conjunction with the direct antifungal activity of AmB, results in superior control of PCM. This study provides a rationale for future research into immunomodulatory strategies as an adjunct to conventional antifungal treatment for PCM.

## Introduction

Paracoccidioidomycosis (PCM) is a systemic infection caused by fungal species of the genus *Paracoccidioides*, including *Paracoccidioides brasiliensis*, *P. lutzii*, *P. americana*, *P. restrepiensis*, and *P. venezuelensis* (Turissini et al., 2017). PCM is endemic to Latin America, with a geographic distribution spanning from Mexico to Argentina, and it is estimated that at least 10 million people are infected (Matute et al., 2006; Marques and Shikanai-Yasuda, 1994). In Brazil, the annual incidence rate ranges from 0.71 to 3.7 cases per 100,000 inhabitants (Martinez, 2017), making it the most prevalent systemic mycosis in the country. The average mortality rate is 1.4 per million per year, ranking PCM as the eighth most frequent cause of death by chronic infectious diseases (Bellissimo-Rodrigues et al., 2011; Coutinho et al., 2015).

Infection is frequently linked to agricultural activities in rural areas, such as soil disturbance and crop transport. PCM has substantial socioeconomic impact, as it predominantly affects agricultural workers and compromises their labor capacity. In fact, spatiotemporal analysis (2014-2023) of acute PCM hospitalizations in Brazil reveals a significant national burden, with 4,232 admissions recorded across 23% of municipalities. The study shows that the rise of acute PCM in Brazil is linked to areas of intense deforestation, largely driven by agricultural expansion, with new hotspots emerging in regions where ecosystem disruption likely increases human exposure to *Paracoccidioides* ssp (Gadêlha et al., 2025). The disease occurs in acute–subacute or chronic forms and affects both immunocompetent and immunocompromised individuals (Marques and Shikanai-Yasuda, 1994). The acute/juvenile form, more common in children and young adults, is associated with a predominant Th2/Th9 response, which impair the formation of compact granulomas and favor pathogen dissemination (Franco et al., 1987). The chronic/adult form, the most frequent presentation, shows a mixed but Th17/Th22-biased response, with contributions from Th1 immunity. This profile confers partial resistance but may also drive excessive inflammation, neutrophil recruitment, tissue damage, and fibrosis (Franco et al., 1987; De Castro et al., 2013). Protective immunity is primarily linked to a strong Th1 response, promoting compact granuloma formation and increased IL-2 and IFN-γ production. In contrast, severe forms are associated with a Th2-dominant profile and poorly organized granulomas (Pagliari et al., 2011). Th17 cells may also participate in host defense, particularly when Th1 mechanisms fail to fully control the infection (Loures et al., 2009; Pagliari et al., 2011).

Several studies have highlighted the significant role of regulatory T cells (Tregs) in PCM development and control (Cacere et al., 2008; Cardoso et al., 2014; Ferreira et al., 2010; Bazan et al., 2015; Felonato et al., 2012; Galdino et al., 2018). In human PCM, Treg numbers correlate with both fungal burden in lesions and disease severity (Cardoso et al., 2014; Cavassani et al., 2006; Ferreira et al., 2010). Additionally, experimental PCM models have advanced understanding of immunoregulation. While Treg depletion enhances Th1/Th17-mediated host resistance, complete absence can trigger excessive immunity and tissue damage (Bazan et al., 2015; Felonato et al., 2012). Depleting Tregs during established PCM, reflecting the clinical scenario of active disease, restored protective Th1/Th17 responses without causing tissue pathology or increasing mortality (Galdino et al., 2018), highlighting potential for immunotherapy in PCM.

Recent advances have been made in PCM treatment, yet significant limitations remain. Common antifungals include cotrimoxazole, triazoles (itraconazole, fluconazole), and amphotericin B. According to both Brazilian and international guidelines, itraconazole (200 mg daily for 9–12 months) is first-line for mild to moderate PCM, with response rates of 85–90% and good tolerability (Shikanai-Yasuda et al., 2017; Thompson et al., 2021; Griffiths et al., 2019), though oral absorption can be variable and intravenous formulations are limited (Andrade et al., 2019). Severe PCM is treated with short-course (2–4 weeks) amphotericin B induction followed by long-term itraconazole (200–400 mg daily) maintenance therapy, but adherence remains a challenge (Thompson et al., 2021). Chronic inflammation and fibrosis may persist post-treatment, affecting quality of life (Griffiths et al., 2019; Thompson et al., 2021). Fluconazole is used in specific cases, such as hepatotoxicity, drug hypersensitivity, or neuro-PCM (Andrade et al., 2019). Management also requires attention to drug interactions and potential adverse effects, including hepatotoxicity, nausea, rash, and electrolyte disturbances (Shikanai-Yasuda, 2015). Given the limitations of current antifungal regimens and their associated treatment burdens, there is a rationale for investigating new therapeutic strategies. A promising approach involves combining immunomodulatory agents designed to restore protective host immunity with direct antifungal action, thereby achieving synergistic control of the infection. Notably, the potential of immune checkpoint inhibition as adjunctive therapy for severe fungal infections has gained clinical traction following reports of spectacular remission in mucormycosis with anti-PD-1 therapy (Grimaldi et al., 2017). These developments underscore the translational relevance of our preclinical investigation.

Monoclonal antibodies (mAbs) are immunotherapeutic agents widely used in the treatment of cancer and immune disorders (Boniche-Alfaro et al., 2021; Patel et al., 2009; Rimawi et al., 2015). Beyond oncology, mAbs show great promise in combating infectious diseases, not only through direct action against pathogens but also by enhancing the efficacy of other antimicrobial drugs against multidrug-resistant microorganisms (Boniche-Alfaro et al., 2021; Preite et al., 2023). A prominent therapeutic application of mAbs is the targeted blockade of co-inhibitory receptors, or immune checkpoints, which can regulate T-cell function. Among inhibitory pathways, those mediated by programmed cell death protein-1 (PD-1/CD279) and cytotoxic T-lymphocyte-associated protein 4 (CTLA-4/CD152) are the most well-characterized and are utilized to counteract T cell exhaustion (Howard et al., 2021; Cox et al., 2017; Kaufmann et al., 2007; Chang et al., 2013). This state of exhaustion arises from the failure to rapidly control and eliminate pathogens or cancer cells, leading to persistent immunopathology and tissue damage. Upon binding to its ligands PD-L1 or PD-L2, which are expressed on various cell types, PD-1 inhibits signaling mediated by the T and B lymphocytes, leading to apoptosis, anergy, and exhaustion (Riella et al., 2012).

Several studies have explored checkpoint inhibitors as a therapeutic strategy for infectious diseases. The use of anti-PD-1/PD-L1 or anti-CTLA-4 antibodies in SIV or HIV infection led to the rapid expansion of virus-specific CD8+ T cells with enhanced functional quality (Velu et al., 2009; Kaufmann et al., 2007). Similar results were observed in toxoplasmosis and leishmaniasis, where PD-1 blockade *in vivo* restored T cell proliferation and function, preventing mortality and even achieving complete resolution of chronic lesions (Bhadra et al., 2011; Mou et al., 2013; Esch et al., 2013). In fungal diseases, studies have demonstrated that checkpoint inhibition can alter the course of the infection. For instance, *Histoplasma capsulatum* induces PD-1 expression on macrophages, which inhibits T lymphocyte proliferation. The absence of PD-1 expression or its blockade with a monoclonal antibody resulted in less severe disease in a murine model (Lázár-Molnár et al., 2008). In models of systemic candidiasis and cryptococcosis, treatment with anti-PD-1 and anti-CTLA-4 led to less severe disease, reduced fungal burden, increased MHC class II molecule expression, and a reinvigorated immune response dominated by protective Th1 lymphocytes secreting IFN-γ (Chang et al., 2013; Roussey et al., 2017).

Building on the established link between PCM-associated immunosuppression and the upregulation of co-inhibitory molecules, in a previous study, we demonstrated that monoclonal antibody-mediated inhibition of CTLA-4 and PD-1 during established pulmonary PCM significantly reduced fungal loads in the lungs and diminished dissemination. This was accompanied by a decrease in pulmonary lesion size and a consequent increase in host survival (Preite et al., 2024a). Immunological analysis revealed that while the total numbers of CD4^+^ and CD8^+^ T cells in the lungs were reduced, consistent with diminished antigenic load and inflammation, the critical populations of Th1, Th2, and Th17 cells were sustained. Notably, this restored immunity controlled fungal growth without exacerbating tissue-damaging inflammation, a critical safety consideration. Given that anti-PD-1 monotherapy reverses T cell exhaustion and amphotericin B provides rapid, potent fungicidal activity in severe PCM, we hypothesized that their combination would be synergistic. This strategy directly parallels the current clinical paradigm for severe PCM, which employs amphotericin B induction followed by long-term itraconazole maintenance (Shikanai-Yasuda, 2015; Griffiths et al., 2019). While effective, this sequential approach presents significant limitations: AmB-related toxicity often necessitates dose reduction, and long-term itraconazole adherence is compromised by gastrointestinal intolerance and variable absorption (Andrade et al., 2019; Peçanha et al., 2016). Critically, neither agent addresses the underlying immune dysfunction, T cell exhaustion, and elevated Treg frequencies that perpetuate chronic inflammation and fibrosis even after apparent microbiological resolution (Ferreira et al., 2010; Cardoso et al., 2014).

Our proposed combination differs fundamentally from conventional regimens. Rather than sequential administration, concurrent anti-PD-1 and AmB enables simultaneous antifungal activity and immune checkpoint blockade, targeting both active infection and the exhausted T cell phenotype that antifungal therapy alone fails to reverse. By restoring protective Th1/Th17 immunity while AmB reduces fungal burden, this approach may achieve more durable disease control, potentially shortening antifungal duration and reducing cumulative toxicity, a strategy increasingly investigated in other chronic infections (Rao et al., 2017).

## Materials and methods

### Animals

Male wildtype C57BL/6 mice, aged 8 to 12 weeks, were used in this study. The animals, originally sourced from Jackson Laboratories, were obtained as specific pathogen-free from the Center for the Development of Experimental Models for Biology and Medicine (CEDEME-UNIFESP). Animals were housed in the animal facility of the Institute of Science and Technology (UNIFESP, São José dos Campos). All experimental procedures involving mice were approved by the UNIFESP Animal Ethics Committee (Protocol No. 8441160424).

### Infection with *P. brasiliensis*

Mice were infected with the virulent Pb18 strain of *P. brasiliensis*, maintained by weekly subculturing in Fava Netto medium at 37 °C. Fungal cells were harvested and washed with phosphate-buffered saline (PBS, pH 7.2). For infection, mice were anesthetized via intraperitoneal (i.p.) injection of ketamine (90 mg/kg) and xylazine (10 mg/kg), followed by intratracheal (i.t.) inoculation with 1×10^6^ yeast cells suspended in 50 μL of PBS, as previously described (Cano et al., 1995). This method ensures direct delivery of fungal cells to the lungs.

### Combined treatment of anti-PD-1 with AmB

Six weeks post-infection, mice were randomly assigned to treatment groups. I.p. injections of anti-PD-1 at a dose of 5 mg/kg (CD279 BioXCell, Cat # BE0146), as based on a prior study (Preite et al., 2024a). The 6-week time point was selected based on our previous characterization of the murine PCM model, which demonstrates that infection is chronically established with persistent fungal burden, organized granulomatous lesions, and T cell exhaustion (Preite et al., 2023, 2024b). Amphotericin B (AmB, with added sodium deoxycholate), 5 mg/kg, was selected as the antifungal agent. This specific formulation and dose were recently established by our group in murine PCM through systematic dose-response testing, demonstrating significant fungal burden reduction across all organs with no acute mortality or severe toxicity (Franco et al., 2025). The placebo control mice group was treated with PBS in the same volume and frequency as the experimental groups. Mice were euthanized at the indicated time points under anesthesia by intraperitoneal (i.p.) injection of ketamine (90 mg/kg) and xylazine (10 mg/kg), followed by cervical dislocation. Three treatment regimens were implemented:

1. Efficacy and immune profiling (8-week endpoint): Treatment for 2 weeks (administered every 48 hours) after 6 weeks of infection, with euthanasia performed after 8 weeks of infection. Mice received PBS, anti-PD-1, AmB, or anti-PD-1+AmB (n=10-11/group for survival; n=4-5/group for CFU, histopathology, ELISA, and flow cytometry).
2. Extended infection (11-week endpoint): Mice received anti-PD-1, AmB, or anti-PD-1+AmB (n=4-5/group for CFU) following two protocols:
  - Single treatment: Treatment for 2 weeks (administered every 48 hours) after 6 weeks of infection, followed by a 3-week treatment-free period, euthanasia at week 11.
  - Prolonged treatment: Treatment for 2 weeks (administered every 48 hours) after 6 weeks of infection, followed by a 1-week interval and a second 2-week treatment, euthanasia at week 11.

Both single treatment and prolonged treatment were also used in a survival test (10 mice per group). The groups used in this experiment were only the combined single and the combined prolonged treatment.

### CFU assays, survival rates, and histological analysis

Viable fungal loads in the lungs, liver, and spleen were quantified by colony-forming unit (CFU) counts, following the established protocol (Singer-Vermes et al., 1993). Survival studies were conducted using groups of 10 mice, with deaths recorded daily. For histopathological evaluation, the lungs and liver were harvested and fixed in 10% formalin. Tissue sections (5 μm) were stained with hematoxylin and eosin (for lesion assessment) and silver stain (for fungal detection). Morphometric analysis was performed using a Nikon DXM 1200c camera and Nikon NIS AR 2.30 software.

### Cytokine detection in lung cell supernatants

The lungs, livers, and spleens of infected animals were removed and individually dissociated in 5 mL of PBS. The supernatants were separated from cell debris by centrifugation at 3,000g for 10 min and stored at -80 °C. Levels of IL-1β, IL-2, IL-4, IL-6, IL-10, IL-12, IL-17, IL-22, IL-23, TNF-α, IFN-γ, and TGF-β were measured by enzyme-linked immunosorbent assay (ELISA) from eBioscience or Biolegends according to the manufacturers’ instructions. Plates were read using a spectrophotometric plate reader (VersaMax, Molecular Devices). For clarity of presentation, cytokines were grouped according to their functional profiles: pro-inflammatory (IL-1β, TNF-α), Th1-associated (IFN-γ, IL-2, IL-12), immunosuppressive (IL-10, TGF-β), Th2-associated (IL-4), and Th17-associated (IL-17, IL-6, IL-22, IL-23).

### Isolation of lung-infiltrating leukocytes

Cell suspensions were prepared as described by Loures et al. (2011). Lungs were harvested and subjected to digestion in digestion buffer containing collagenase (2 mg/mL) and DNase (1 mg/mL) for 40 minutes at 37 °C with constant agitation. The tissues were then mechanically dissociated using a glass homogenizer in RPMI 1640 culture medium (Sigma-Aldrich) to obtain a cell suspension, which was subsequently filtered through a 70 μm cell strainer (Falcon) to ensure proper cell separation. Cells were counted using a hemocytometer, and viability was assessed by Trypan Blue.

### Flow cytometry assay

Myeloid cells and lymphocyte subpopulations were analyzed using multiparametric flow cytometry. Cells isolated from the lungs of infected animals were stained for total leukocytes (CD45^+^), myeloid cells (CD45^+^CD11b^+^), dendritic cells (CD45^+^CD11b^+^CD11c^+^F4/80^−^), alveolar macrophages (CD45^+^CD11b^+^F4/80^+^), and neutrophils (CD45^+^CD11b^+^Ly6G^hi^). Additionally, dendritic cells and macrophages were assessed for CD40, CD80, CD86, and MHC class II expression. Populations of CD4^+^ and CD8^+^ T lymphocytes, along with their activation markers, CD25 and CD69, respectively, were also analyzed. Cell suspensions were adjusted to a concentration of 1×10^6^ cells/well in U-bottom plates. Cells were resuspended in PBS containing 0.1% sodium azide and 2% fetal bovine serum (FBS). After centrifugation and supernatant removal, cells were incubated with fluorochrome-conjugated antibodies following Fc receptor blockade with anti-CD16/32 monoclonal antibody, with a final volume of 25 μL/well at appropriate titers. Following incubation at 4 °C, plates were washed twice with PBS-azide, and cells were resuspended in 200 μL of 2% paraformaldehyde for acquisition. All samples were kept on ice and protected from light. A minimum of 50,000 events were acquired on a FACSLyric flow cytometer (BD Biosciences) using FACSuite software (BD Biosciences). Analysis of surface marker expression was performed using FlowJo software (Tree Star). The gating strategy for myeloid cells is detailed in the **Supplementary Figure 1**. Flow cytometry data are presented as both frequency (percentage of the indicated population) and absolute number (total cells per lung, calculated by multiplying the frequency by the total viable cell count). Frequency reflects the relative enrichment of a population, while absolute numbers indicate the total cellular load in the organ. These complementary measures provide insight into both the composition and magnitude of the pulmonary immune response.

### Intracellular staining in lymphocytes

For lymphocyte populations, intracellular staining for IFN-γ (Th1 and Tc1), IL-4 (Th2 and Tc2), and IL-17 (Th17 and Tc17) was performed in CD4^+^ and CD8^+^ T lymphocytes by flow cytometry. The presence of the transcription factor Foxp3 (Treg) was also analyzed in CD4^+^CD25^+^ T cells. Lung samples were resuspended at 1×10^6^ cells/mL and stimulated with PMA (50 ng/mL), ionomycin (500 ng/mL), and brefeldin A (1:1000) for 6 hours to enable cytokine accumulation. Following stimulation, cells were surface-stained with anti-CD4, CD8, CD69, and CD25 antibodies. After permeabilization using the Cytofix/Cytoperm Plus kit (BD Biosciences), intracellular staining was performed with anti-IFN-γ, IL-4, IL-17, and FoxP3 antibodies according to the manufacturer’s specifications. Cells were analyzed on a FACSLyric flow cytometer, and population analysis was conducted using FlowJo software (Tree Star). The gating strategy for the lymphocyte population is detailed in the **Supplementary Figure 2**.

### Statistical analysis

Data were initially assessed for normal distribution and homogeneity of variances using the F-test. Parametric tests (Student’s t-test, one-way or two-way ANOVA) were applied to normally distributed data with homogeneous variances, followed by Bonferroni *post-hoc* testing. Non-parametric data were analyzed using the Mann-Whitney test. Survival curves were compared using the Log-rank (Mantel-Cox) test. Results are expressed as mean ± standard deviation (SD). Statistical analyses and graphing were performed with GraphPad Prism 9.0. To quantify the magnitude of treatment effects after CFU results, Cohen’s d effect sizes were calculated for pairwise comparisons using the formula: d = (M_1_ – M_2_)/SDpooled, where SDpooled = √[(SD_1_^2^ + SD_2_^2^)/2]. Effect sizes were interpreted as small (0.2), medium (0.5), large (≥0.8), very large (≥1.5), or huge (≥2.0) according to conventional thresholds (Cohen, 1988). All calculations were performed on log_10_-transformed CFU data using GraphPad Prism 9.0 and Microsoft Excel. Results are presented with 95% confidence intervals.

## Results

### Combined anti-PD-1 and amphotericin B therapy reduces fungal burden and enhances control of Murine PCM

**Figure 1:**
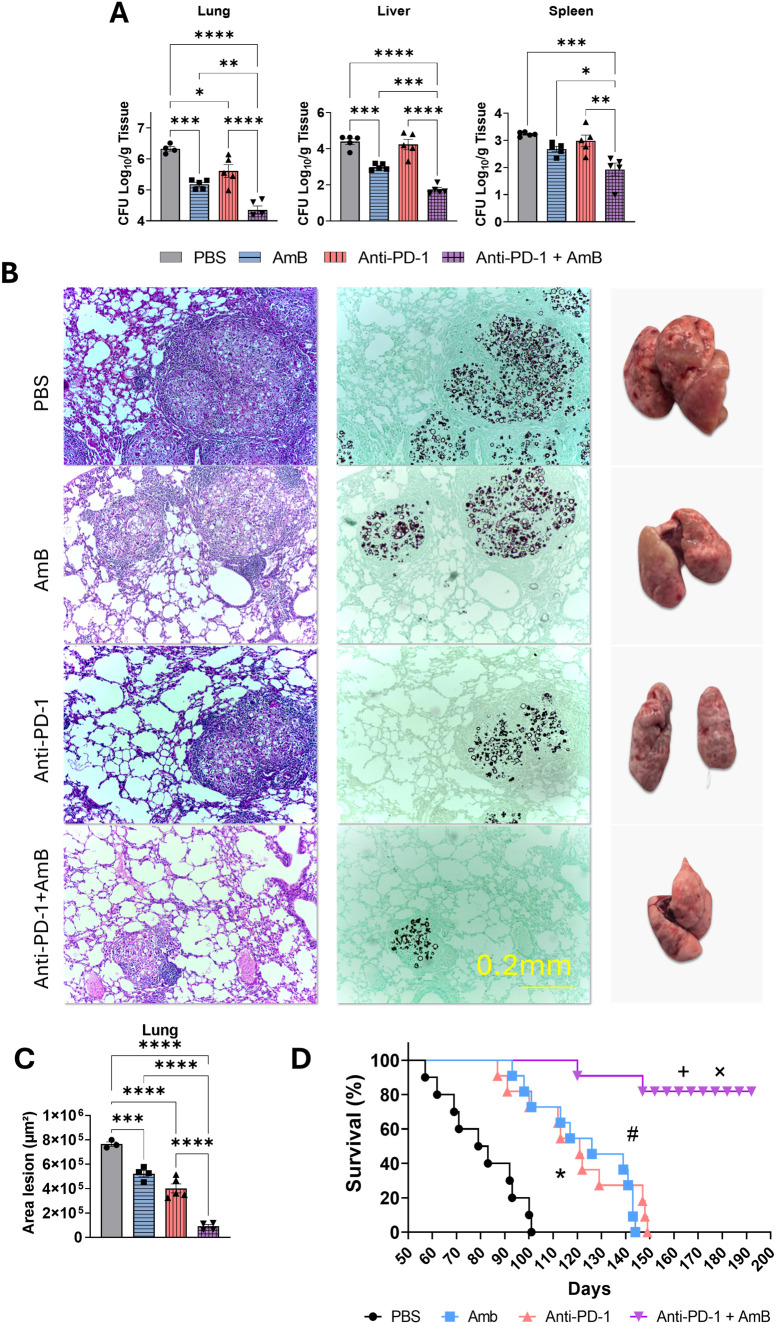
Combined anti-PD-1 and amphotericin B therapy reduces fungal burden and improves survival in murine paracoccidioidomycosis (PCM). C57BL/6 mice were infected intratracheally (i.t.) with 1 × 10^6^ *P. brasiliensis* yeasts. After 6 weeks, treatment began with intraperitoneal (i.p.) injections of amphotericin B (AmB; 5 mg/kg), anti–PD-1 (5 mg/kg), the combination (Anti–PD-1 + AmB), or PBS vehicle control on alternate days for 2 weeks. **(A)** Colony-forming units (CFU) in the lungs, liver, and spleen were determined at week 8 post-infection. Data are pooled from three independent experiments (n=4–5 mice per group per experiment). Bars represent mean ± standard error, analyzed by analysis of variance (ANOVA). *p <0.05; **p <0.01; ***p <0.001 and ****p <0.0001. **(B)** Representative lung histology (left panels) and gross lung morphology (right panels). Lung sections were stained with hematoxylin and eosin (H&E; left panels) to assess inflammation or by the Grocott method (methenamine silver staining; right panels) to visualize fungal elements. **(C)** The total area of lung lesions was calculated in square micrometers of 5 microscopic fields per slide. Comparisons between two groups, mean ± standard deviation (SD), were analyzed by ANOVA. ***p <0.001 and ****p <0.0001. **(D)** Mice (n=10–11 per group) were infected and treated as described above and monitored for survival. Data are from a single experiment. Statistical comparisons were made using the log-rank (Mantel–Cox) test. PBS vs. AmB, p < 0.0001 (#); PBS vs. Anti–PD-1, p < 0.0002 (*); Anti–PD-1+AmB vs. AmB, p < 0.0001 (+); Anti–PD-1+AmB vs. Anti–PD-1, p < 0.0001 (×).

In a previous study published by our group, anti-PD-1 monotherapy was shown to reduce fungal burden in the lungs, liver, and spleen of infected animals (Preite et al., 2024a). Here, we evaluated whether combined treatment (anti-PD-1 + AmB) could enhance the effects observed with either therapy alone. C57BL/6 mice were infected for eight weeks and received different treatments every 48 hours during the final two weeks. Following sacrifice, lungs, livers, and spleens were harvested for fungal recovery and plated on BHI agar for colony counting. As shown in **Figures 1A**, the combined treatment significantly reduced the fungal burden, as measured by colony counts, in all organs analyzed. Treatment with AmB or anti-PD-1 alone also significantly reduced the pulmonary fungal load compared to PBS-treated mice. In the liver, AmB monotherapy, but not anti-PD-1 alone, resulted in a significant reduction in CFU compared with PBS treatment. Notably, in the spleen, neither monotherapy achieved a significant reduction in fungal burden, a statistically significant decrease was observed exclusively in the group receiving the anti-PD-1 and AmB combination.

Representative micrographs of Hematoxylin and Eosin (H&E, for tissue morphology) and Grocott (for fungal visualization) stained sections from lungs are shown in **Figure 1B** (H&E left and Grocott right panels). These images confirmed the improved tissue preservation in the anti-PD-1+AmB treatment group. The combination treatment provided superior protection, characterized by minimal granulomatous lesions that were more compact and defined, along with thinner alveolar walls and reduced cellular infiltration. Animals receiving anti-PD-1 or amphotericin B showed reduced size of granulomatous lesions and less compromised tissue compared to PBS-treated mice. Also, the macroscopic photos of the lungs revealed a better-preserved aspect in animals treated with anti-PD-1 and amphotericin B compared to controls. Morphometric lesion analysis confirmed significantly less pulmonary tissue involvement in the combination group compared to individual treatments (**Figure 1C**). While individual treatments also reduced the affected area compared to PBS controls, as we previously demonstrated (Preite et al., 2024a), the combination was significantly more effective.

To evaluate the long-term effect of combined treatment, first we conducted survival analysis with treatments administered from week 7 until the end of week 8. As shown in **Figures 1D**, the combination of anti-PD-1 and AmB conferred a significant survival advantage, with over 80% of mice surviving to the experimental endpoint. This group experienced only two fatalities, with the first death occurring 120 days post-infection. In contrast, both anti-PD-1 and amphotericin B monotherapies, while improving survival compared to the PBS treatment, were less effective. The PBS-treated group exhibited the first mortality at 60 days and reached 100% mortality by day 100 post-infection. The monotherapy groups showed delayed but ultimately complete mortality, with initial deaths occurring between days 90–100 and 100% mortality between days 140-150.

To quantify the therapeutic improvement conferred by the anti-PD-1 + AmB combination, we calculated Cohen’s d effect sizes for all pairwise treatment comparisons (**Supplementary Table 1**). The combination therapy produced huge reductions in fungal burden compared to PBS control (d = –9.74 [95% CI: –13.44 to –6.04]), AmB monotherapy (d = –5.38 [–7.59 to –3.17]), and anti-PD-1 monotherapy (d = –3.27 [–5.12 to –1.42]). These values far exceed the conventional threshold for a large effect (d ≥ 0.8) and demonstrate that the superiority of the combination is not only statistically significant but also clinically meaningful. Complete effect sizes with 95% confidence intervals are presented in **Supplementary Table 1**.

### Prolonged treatment improves the therapeutic effect of combined anti-PD-1 and amphotericin B

**Figure 2:**
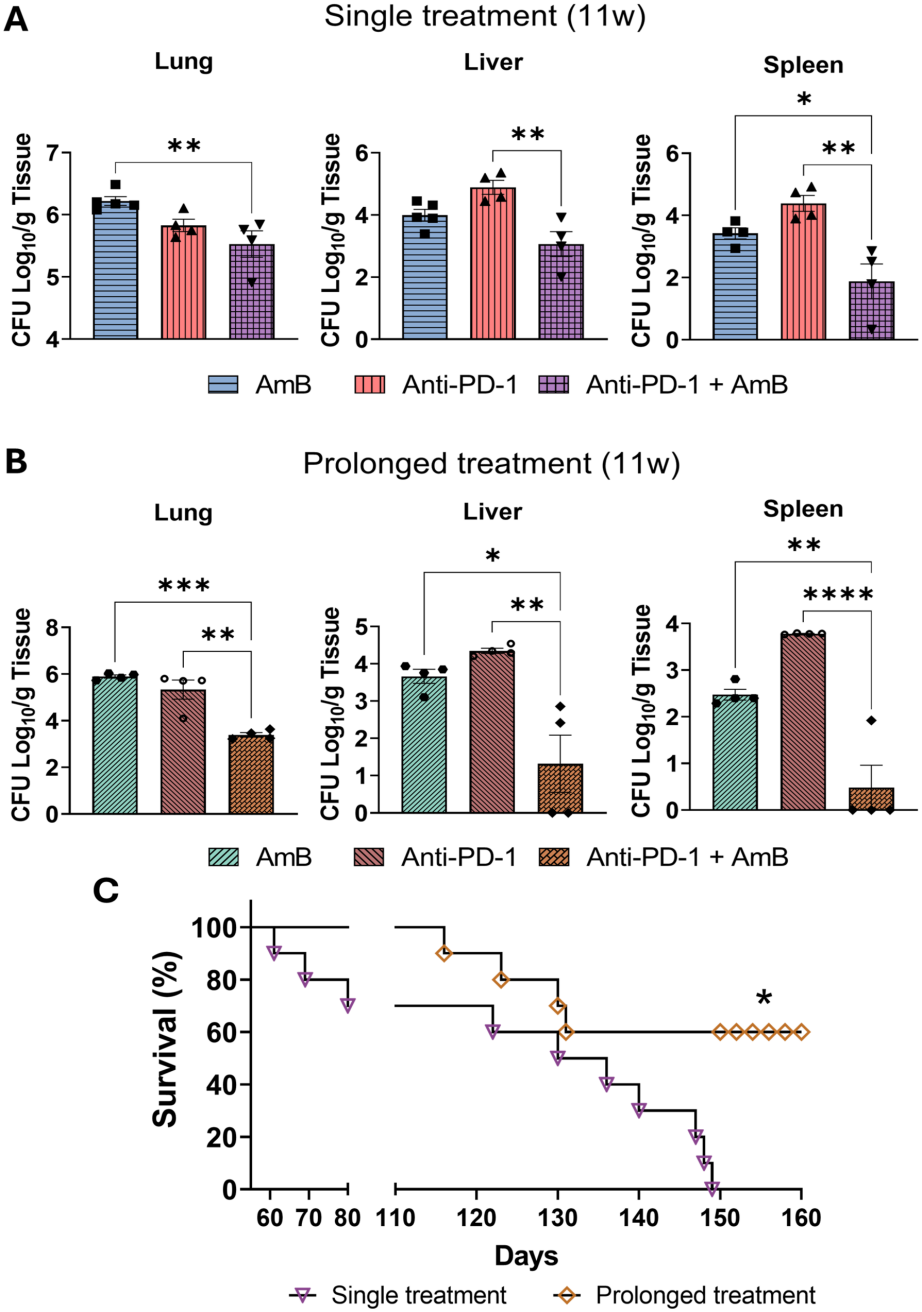
Prolonged combination therapy achieves superior long-term efficacy compared to a single treatment course. C57BL/6 mice were infected intratracheally (i.t.) with 1 × 10^6^ *P. brasiliensis* yeasts. After 6 weeks, both single and prolonged treatment began with intraperitoneal (i.p.) injections of amphotericin B (AmB; 5 mg/kg), anti–PD-1 (5 mg/kg), the combination (Anti–PD-1 + AmB), or PBS vehicle control on alternate days for 2 weeks. Animals in the single-treatment group received no further i.p. treatments. For the prolonged treatment regimen, after the initial 2-week course and a subsequent 1-week pause, an identical second 2-week course was administered. **(A, B)** Colony-forming units (CFU) in the lungs, liver, and spleen were determined at week 11 post-infection for both single **(A)** and prolonged **(B)** treatments. Data are pooled from two independent experiments (n=4–5 mice per group per experiment). Bars represent mean ± standard deviation (SD), analyzed by analysis of variance (ANOVA). *p <0.05; **p <0.01; ***p <0.001 and ****p <0.0001. **(C)** Mice (n=10 per group) were infected and treated as described above and monitored for survival. Data are from a single experiment. Statistical comparisons were made using the log-rank (Mantel–Cox) test. p < 0.05.

Given the observed reduction in fungal burden, improved tissue preservation, and enhanced survival with combination therapy, we investigated whether these benefits would persist after treatment cessation. We extended the infection period to 11 weeks, with treatments starting from week 7 to the end of week 8, followed by three treatment-free weeks. This design allowed us to determine: (1) durability of the protective effect; (2) whether disease regression would continue; and (3) if combination therapy could achieve complete fungal clearance in target organs. Even after 11 weeks of infection, with the last three weeks without treatment, combined anti-PD-1 and AmB therapy significantly reduced lung CFU compared to amphotericin B monotherapy. Similarly, in the liver, combination therapy showed greater reduction compared to anti-PD-1 alone. In the spleen, the combination treatment resulted in superior CFU reduction compared to both individual therapies (**Figure 2A**). However, while the protective effect of combination therapy persisted, the fungal burden not only remained detectable but showed resurgence compared to 8-week levels, indicating interrupted disease regression despite initial treatment success. Based on these results, we established an extended treatment strategy consisting of two 2-week therapeutic cycles separated by a 1-week rest period, aiming to achieve sustained disease regression (**Figure 2B**). The extended combination of anti-PD-1 + AmB proved superior, resulting in significantly lower CFU counts than prolonged individual treatments. Notably, only this prolonged combined regimen achieved fungal burdens lower than those recorded after any single treatment, whether assessed at the 8-week or 11-week postinfection. Finally, the survival benefit of the extended regimen was evaluated. Animals that received the prolonged combined treatment (two cycles) demonstrated a significantly higher survival rate compared to those receiving only a single cycle of combination therapy, underscoring that the long-term efficacy of anti-PD-1 + AmB is further enhanced by sustained treatment (**Figure 2C**).

### Combined anti-PD-1 and amphotericin B therapy modulates inflammatory responses in murine PCM

When developing new therapies, especially those that modulate the immune system, such as monoclonal antibody-based immune checkpoint inhibitors, it is essential to evaluate both the protective and pathological aspects of the inflammatory response. An effective therapy should promote pathogen clearance while minimizing tissue damage caused by uncontrolled cytokine production or excessive immune cell activation, which can result from checkpoint receptor blockade. To further elucidate the impact of treatment on the local immune response, we quantified cytokine production in organ homogenates from animals infected for eight weeks and treated during the final two weeks.

**Figure 3:**
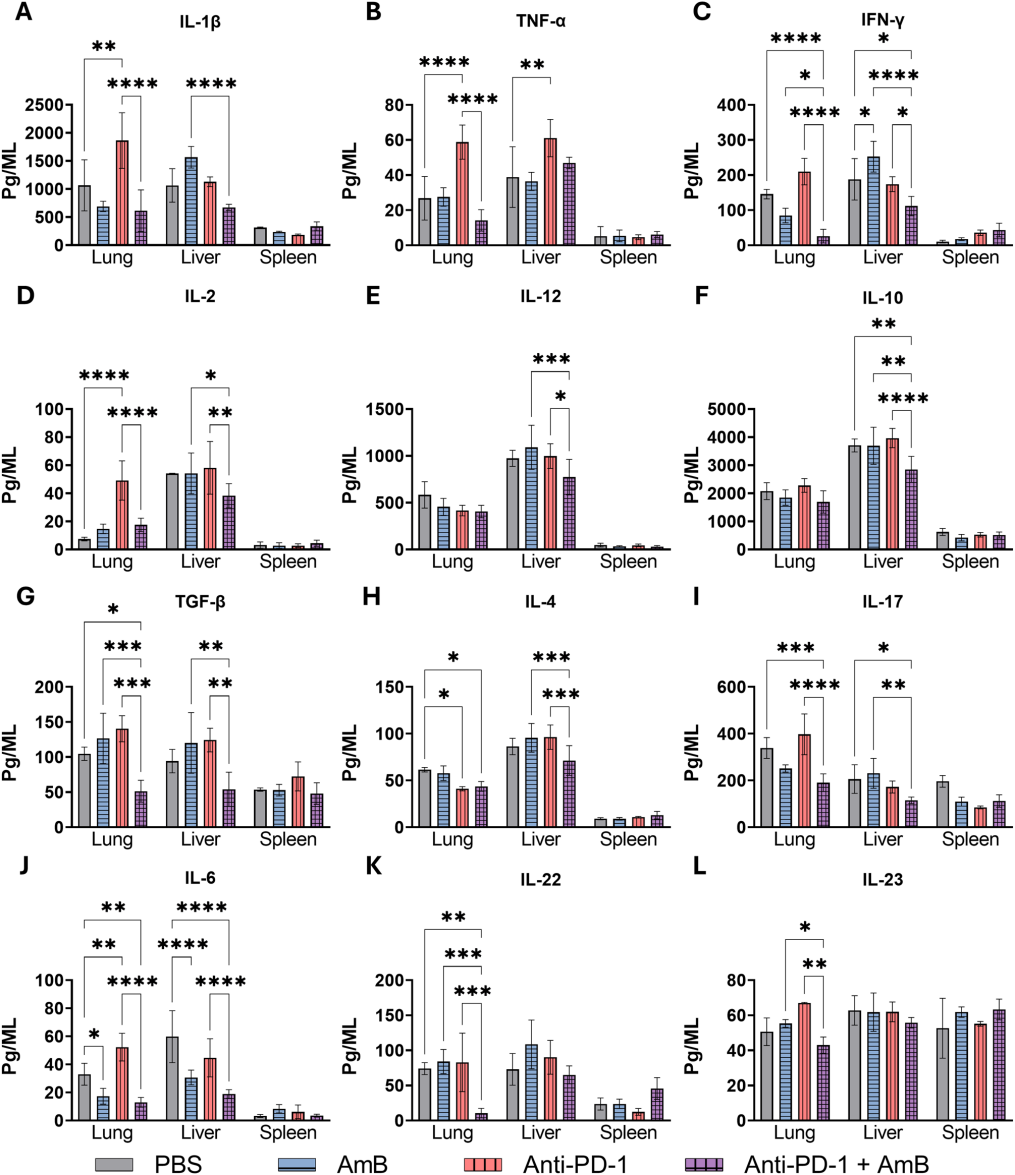
Combined therapy with anti-PD-1 and amphotericin B modulates cytokine profiles in target organs during PCM. C57BL/6 mice were infected intratracheally (i.t.) with 1 × 10^6^ *P. brasiliensis* yeasts. After 6 weeks, treatment began with intraperitoneal (i.p.) injections of amphotericin B (AmB; 5 mg/kg), anti–PD-1 (5 mg/kg), the combination (Anti–PD-1 + AmB), or PBS vehicle control on alternate days for 2 weeks. Lung, liver, and spleen homogenates were obtained, and cytokines were quantified by enzyme-linked immunosorbent assay (ELISA). **(A)** IL-1β, **(B)** TNF-α, **(C)** IFN-γ, **(D)** IL-2, **(E)** IL-12, **(F)** IL-10, **(G)** TGF-β, **(H)** IL-4, **(I)** IL-17, **(J)** IL-6, **(K)** IL-22, and **(L)** IL-23. Data represent three experiments conducted with 4–5 mice each. Bars represent mean ± standard error, analyzed by analysis of variance (ANOVA). *p <0.05; **p <0.01; ***p <0.001 and ****p <0001.

Levels of the pro-inflammatory cytokines IL-1β and TNF-α are shown in **Figures 3A, B**. The combination treatment significantly reduced IL-1β and TNF-α in the lungs compared to anti-PD-1 monotherapy, and IL-1β in the liver compared to AmB treatment. In contrast, anti-PD-1 monotherapy increased IL-1β and TNF-α in the lungs and TNF-α in the liver. No significant differences were observed for these cytokines in the spleen.

Th1-associated cytokines (IFN-γ, IL-2, and IL-12) are shown in **Figures 3C–E**. IFN-γ decreased in the lungs following combination treatment compared to individual therapies and PBS controls, with a similar reduction observed in the liver. Amphotericin B monotherapy, however, increased hepatic IFN-γ compared to the PBS control (**Figure 3C**). IL-2 was elevated in lung homogenates from the anti-PD-1 group compared to the PBS control, an effect that was absent in the combination group (**Figure 3D**). In the liver, IL-2 was reduced in the combination group compared to both monotherapies. Hepatic IL-12 levels were also lower following combination treatment compared to individual therapies (**Figure 3E**). No significant differences were observed in splenic concentrations of IFN-γ, IL-2, or IL-12.

For immunosuppressive cytokines such as IL-10 and TGF-β, as well as the Th2-associated cytokine IL-4, the combination treatment reduced hepatic levels of IL-10 compared to all other groups (anti-PD-1, AmB, and PBS) and also lowered TGF-β levels compared to both monotherapies. (**Figures 3F, G**). A similar reduction in TGF-β was observed in the lungs after the combined treatment. For IL-4, anti-PD-1 treatment, both combined and in single treatment, reduced pulmonary levels compared to the PBS control. In the liver, only the combination treatment significantly decreased IL-4 compared to monotherapy (**Figure 3H**).

The cytokines IL-17, IL-23, and IL-6 are associated with the Th17 profile, while IL-22 is related to tissue repair in PCM (Tristão et al., 2017; De Araújo et al., 2017). In the lungs, combined therapy resulted in a more pronounced suppression of this cytokine profile, significantly reducing IL-17 and IL-6 compared to the anti-PD-1 and PBS groups, while IL-22 was reduced relative to all monotherapies and the control (**Figures 3I–K**). In contrast, the effects of monotherapies were less marked: compared with PBS-treatment, amphotericin B monotherapy reduced IL-6 levels, while anti-PD-1 treatment increased IL-6 (**Figure 3J**). This suppression is consistent with more effective infection control, reducing the need for sustained inflammatory recruitment and activation. In the liver, combination treatment decreased IL-17 levels compared to amphotericin B alone or PBS treatments, and reduced IL-6 compared to anti-PD-1 monotherapy and control. Amphotericin B treatment also reduced hepatic IL-6 compared to the PBS control. Furthermore, combination treatment decreased IL-23 levels in the lungs compared to both monotherapies (**Figure 3L**). In the spleen, none of the therapeutic interventions significantly altered the levels of IL-17, IL-6, IL-22, or IL-23.

### Combined anti-PD-1 and amphotericin B therapy enhances myeloid cell infiltration in the lungs of *P. brasiliensis*-infected mice

**Figure 4:**
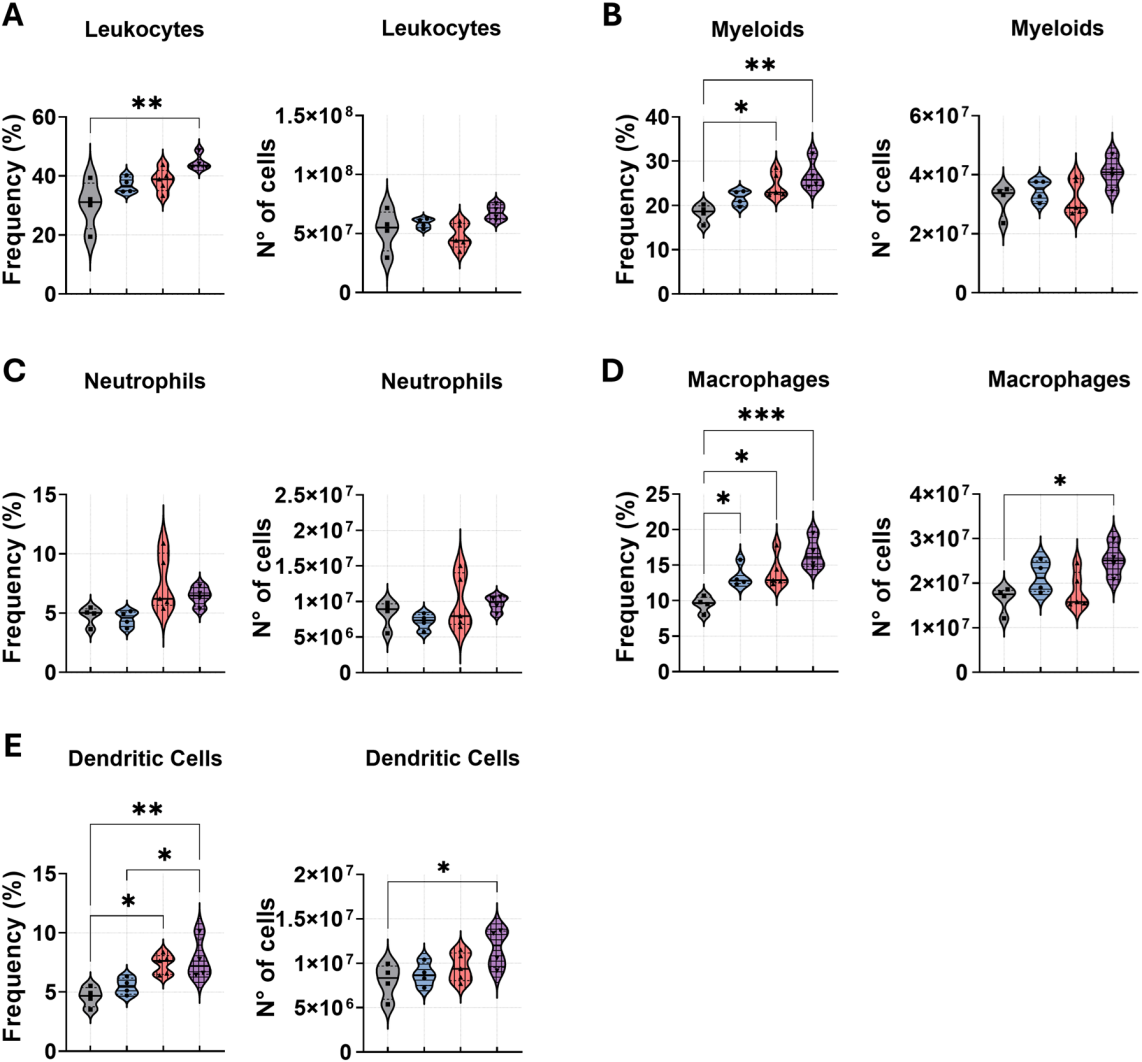
Anti-PD-1 treatment in combination with amphotericin B increases macrophage and dendritic cell populations in the lungs during *P. brasiliensis* infection. C57BL/6 mice were infected intratracheally (i.t.) with 1 × 10^6^ *P. brasiliensis* yeasts. After 6 weeks, treatment began with intraperitoneal (i.p.) injections of amphotericin B (AmB; 5 mg/kg), anti–PD-1 (5 mg/kg), the combination (Anti–PD-1 + AmB), or PBS vehicle control on alternate days for 2 weeks. Frequency and absolute number of **(A)** total leukocytes (CD45^+^), **(B)** total myeloid cells (CD45^+^CD11b^+^), **(C)** neutrophils (CD45^+^CD11b^+^Ly6G^hi^), **(D)** macrophages (CD45^+^CD11b^+^F4/80^+^), and **(E)** dendritic cells (CD45^+^CD11b^+^CD11c^+^F4/80^−^) were analyzed after 8 weeks of infection. Dead cells were excluded using a Live/Dead antibody. Gating strategies are described in **Supplementary Figure 1**. Data from 3 independent experiments, with 3–4 mice per group. Bars represent mean ± standard error, analyzed by analysis of variance (ANOVA). *p < 0.05, **p < 0.01, and ***p < 0.001.

To assess the impact of combined treatment on myeloid and leukocyte dynamics during *P. brasiliensis* infection, we analyzed lung-infiltrating immune cells from C57BL/6 mice through a multiparametric flow cytometry technique. Leukocytes and myeloid cells were identified as CD45^+^ and CD45^+^CD11b^+^ populations, respectively (**Figures 4A, B**). The complete gating strategy for these and subsequent populations is detailed in the supplementary section. Our analysis revealed that anti-PD-1 in combination with AmB significantly increased the frequency of total leukocytes and myeloid cells in the lungs compared to the PBS control. Anti-PD-1 monotherapy also elevated the frequency of myeloid cells. However, the absolute numbers of these populations remained comparable across all treatment groups (**Figures 4A, B**).

**Figure 5:**
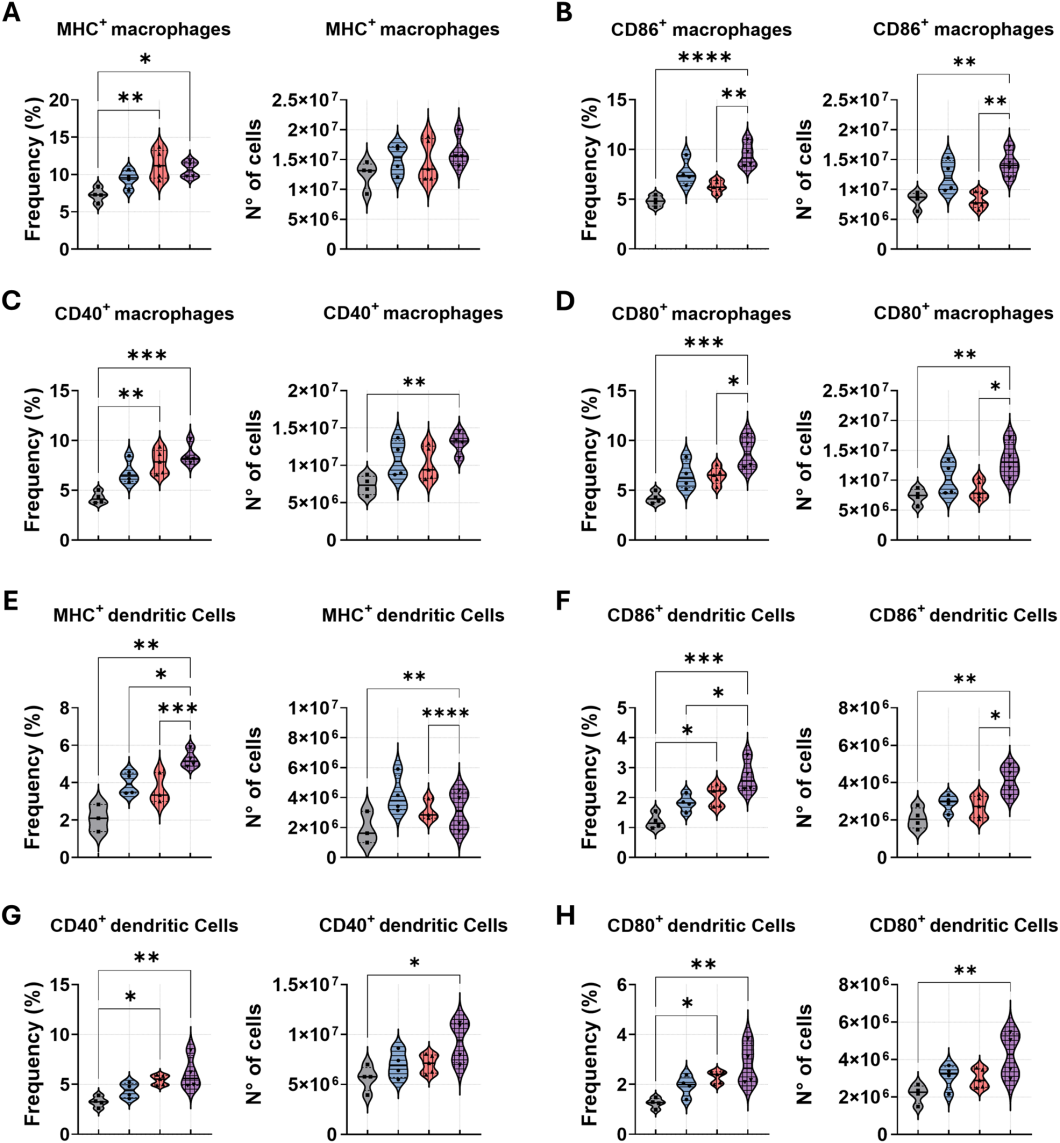
Combined therapy upregulates costimulatory and antigen-presentation molecules on pulmonary macrophages and dendritic cells during *P. brasiliensis* infection. C57BL/6 mice were infected intratracheally (i.t.) with 1 × 10^6^ *P. brasiliensis* yeasts. After 6 weeks, treatment began with intraperitoneal (i.p.) injections of amphotericin B (AmB; 5 mg/kg), anti–PD-1 (5 mg/kg), the combination (Anti–PD-1 + AmB), or PBS vehicle control on alternate days for 2 weeks. Lung-infiltrating cells were analyzed by flow cytometry at week 8 post-infection. **(A–D)** Frequency and absolute number of macrophages (CD45^+^CD11b^+^F4/80^+^) expressing MHC-II **(A)**, CD86 **(B)**, CD40 **(C)**, and CD80 **(D)**. **(E–H)** Frequency and absolute number of dendritic cells (CD45^+^CD11b^+^CD11c^+^F4/80^−^) expressing MHC-II **(E)**, CD86 **(F)**, CD40 **(G)**, and CD80 **(H)**. Gating strategies are described in **Supplementary Figure 1**. Data from 3 independent experiments, with 3–4 mice per group. Bars represent mean ± standard error, analyzed by analysis of variance (ANOVA). *p < 0.05, **p < 0.01, ***p < 0.001, and ****p < 0.0001.

We further investigated key myeloid populations critical for PCM pathogenesis: neutrophils, macrophages, and dendritic cells (DCs) (**Figures 4C–E**). No significant differences were observed in the frequency or absolute numbers of neutrophils across the different treatments (**Figure 4A**). The combination therapy produced the most pronounced effect on macrophages, significantly increasing both their frequency and absolute number compared to the PBS-treated group. The frequency of macrophages was also elevated in both the anti-PD-1 and AmB monotherapy groups relative to the PBS control, though their absolute numbers were not significantly altered (**Figure 4D**). For dendritic cells, the combination treatment significantly elevated their frequency and number compared to the PBS group, however, when compared to AmB monotherapy, only the frequency was significantly elevated. Anti-PD-1 monotherapy also increased DC frequency relative to the PBS control, but did not alter absolute numbers (**Figure 4E**). Surface marker analysis revealed a significant increase in the frequency of MHC class II^+^ and CD40^+^ macrophages in the lungs of mice treated with anti-PD-1, either in combination with AmB or as a monotherapy (**Figures 5A, C**). Critically, only the combination therapy significantly enhanced the frequency of CD86^+^ and CD80^+^ macrophages relative to both anti-PD-1 monotherapy and PBS controls (**Figures 5B, D**). This higher frequency was related to absolute cell counts; the combination group exhibited elevated numbers of CD86^+^/CD80^+^ and CD40^+^ macrophages compared to the PBS control, and a higher number of CD86^+^/CD80^+^ macrophages than the anti-PD-1 monotherapy group. A similar analysis of dendritic cell (DC) populations demonstrated that anti-PD-1 treatment, both combination and monotherapy, increased the frequency of CD40^+^ and CD80^+^/CD86^+^ DCs compared to PBS-treated mice (**Figures 5F–H**). However, a significant increase in the absolute numbers of these activated DC subsets was exclusive to the combination treatment group. Furthermore, the frequency of CD86^+^ DCs was higher following combination therapy compared to amphotericin B alone. The combined regimen also resulted in a higher frequency of MHC-II^+^ DCs than all other treatments and a greater absolute number compared to both the anti-PD-1 and PBS-treated groups (**Figure 5E**).

### Anti-PD-1 synergizes with amphotericin B to enhance CD4^+^/CD8^+^ T cell responses in pulmonary PCM

**Figure 6:**
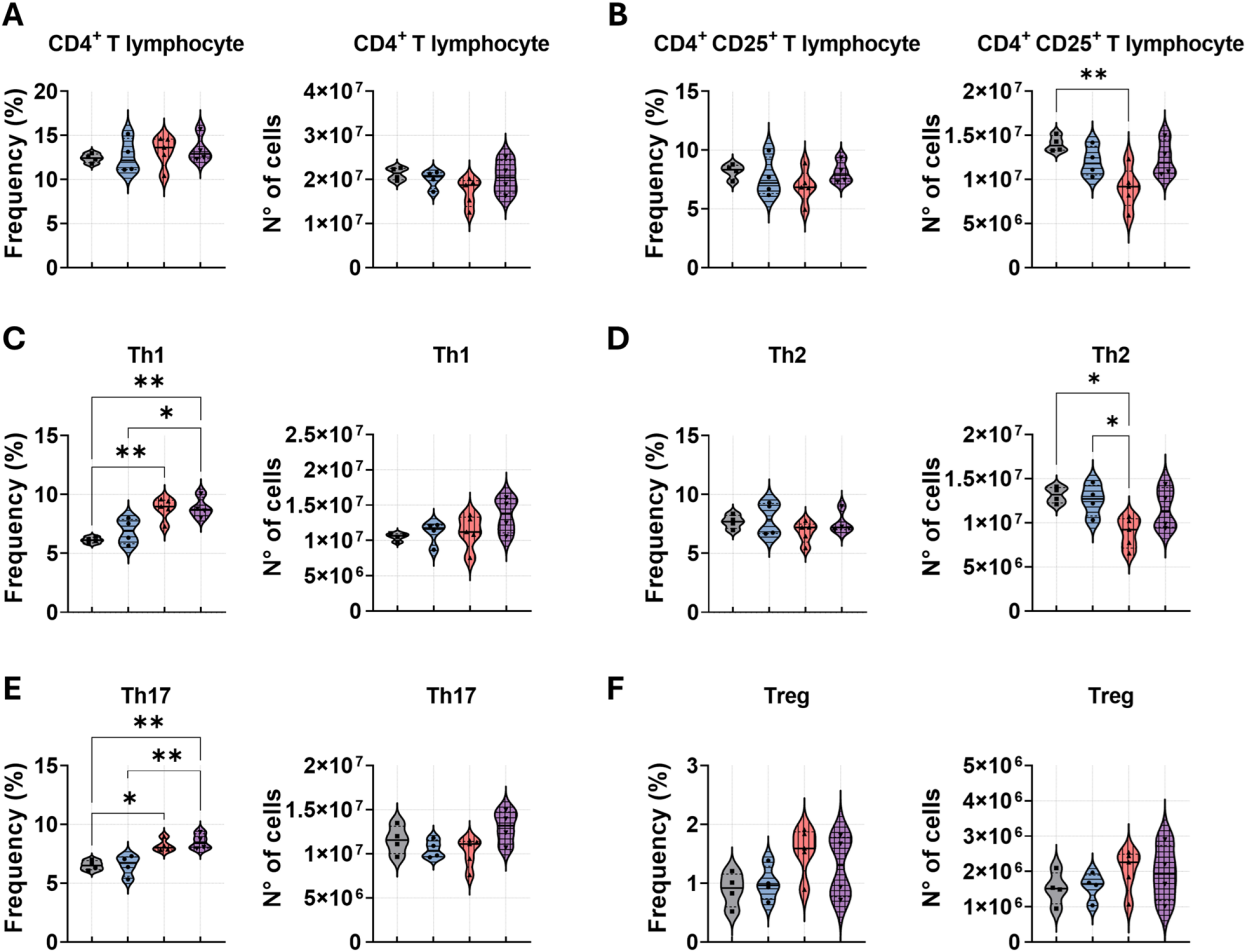
Combined anti-PD-1 and amphotericin B therapy amplifies T-helper 1 and T-helper 17 responses in the lungs during PCM. C57BL/6 mice were infected intratracheally (i.t.) with 1 × 10^6^ *P. brasiliensis* yeasts. After 6 weeks, treatment began with intraperitoneal (i.p.) injections of amphotericin B (AmB; 5 mg/kg), anti–PD-1 (5 mg/kg), the combination (Anti–PD-1 + AmB), or PBS vehicle control on alternate days for 2 weeks. Lung-infiltrating cells were analyzed by flow cytometry at week 8 post-infection. Frequency and absolute number of **(A)** total CD4^+^ T cells, **(B)** activated CD4^+^CD25^+^ T cells, **(C)** T helper 1 (Th1; CD4^+^IFN-γ^+^), **(D)** T helper 2 (Th2; CD4^+^IL-4^+^), **(E)** T helper 17 (Th17; CD4^+^IL-17^+^), and **(F)** regulatory T cells (Treg; CD4^+^CD25^+^Foxp3^+^). Gating strategies are described in **Supplementary Figure 2**. Data from 3 independent experiments, with 3–4 mice per group. Bars represent mean ± standard error, analyzed by analysis of variance (ANOVA). *p < 0.05, and **p < 0.01.

The final flow cytometry analysis assessed the impact of combination therapy on T lymphocyte subsets. Analysis of the CD4^+^ T lymphocyte population revealed that neither the combination treatment nor the monotherapies significantly altered the overall frequency or absolute number of these cells in the infected lungs. An exception was a reduction in the number of CD4^+^CD25^+^ T cells following anti-PD-1 monotherapy compared to the PBS control (**Figures 6A, B**). Further characterization of helper T cell subsets demonstrated that combination treatment increased the frequency of Th1 and Th17 cells compared to both amphotericin B monotherapy and PBS controls (**Figures 6C, E**). The anti-PD-1 treatment similarly elevated the frequencies of these subsets compared to the PBS group. In contrast, anti-PD-1 monotherapy decreased the absolute number of Th2 cells compared to both amphotericin B and PBS treatments (**Figure 6D**). Similarly to the results for the total CD4^+^ population, no significant differences were observed in the frequency or number of regulatory T cells (Tregs) across the treatment groups (**Figure 6F**).

**Figure 7:**
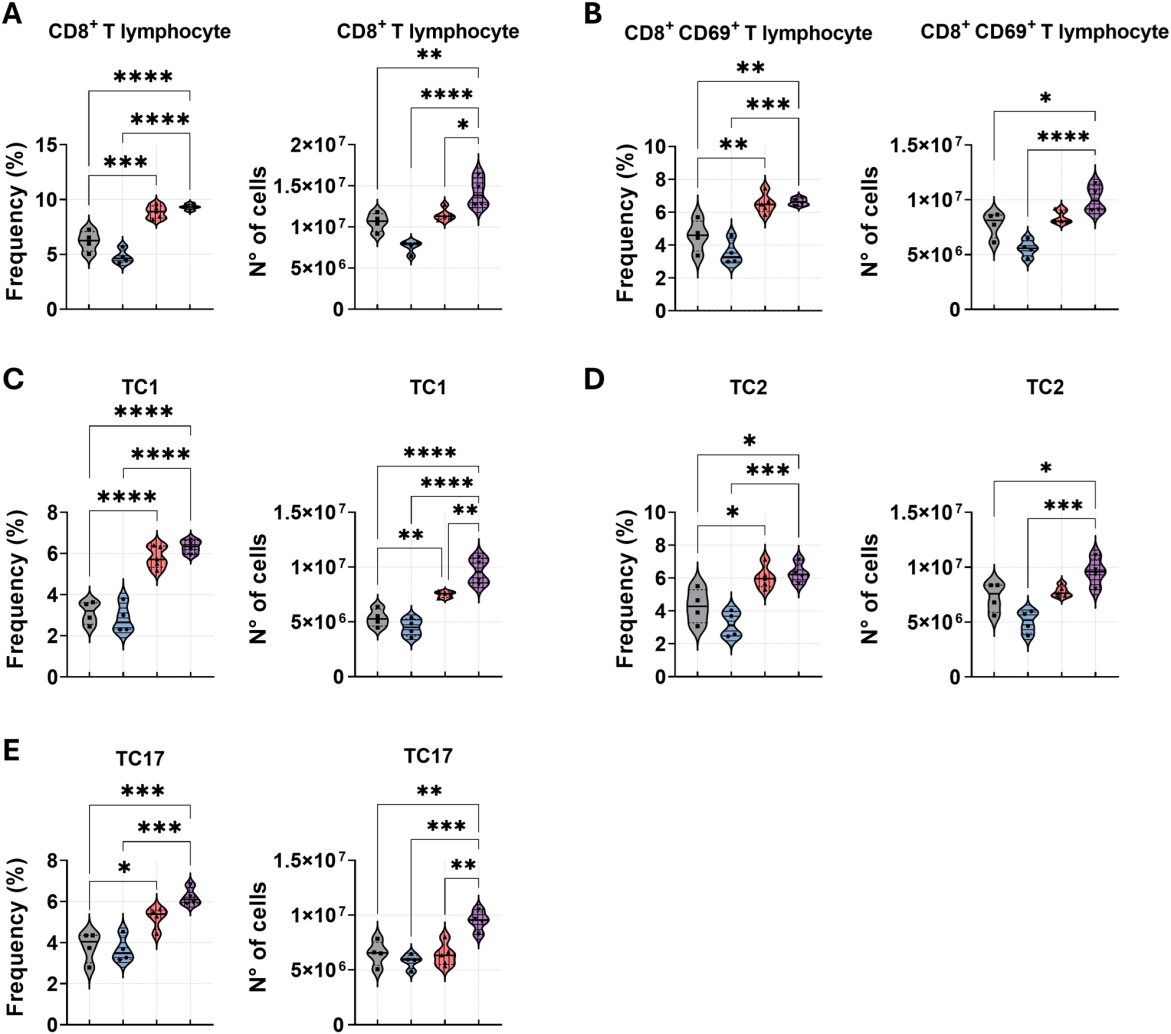
Combined anti-PD-1 and amphotericin B therapy amplifies CD8^+^ T cell and T cytotoxic responses against *P. brasiliensis* infection. C57BL/6 mice were infected intratracheally (i.t.) with 1 × 10^6^ *P. brasiliensis* yeasts. After 6 weeks, treatment began with intraperitoneal (i.p.) injections of amphotericin B (AmB; 5 mg/kg), anti–PD-1 (5 mg/kg), the combination (Anti–PD-1 + AmB), or PBS vehicle control on alternate days for 2 weeks. Lung-infiltrating cells were analyzed by flow cytometry at week 8 post-infection. Frequency and absolute number of **(A)** total CD8^+^ T cells, **(B)** activated CD8^+^CD69^+^ T cells, **(C)** cytotoxic T type 1 (Tc1; CD8^+^IFN-γ^+^), **(D)** cytotoxic T type 2 (Tc2; CD8^+^IL-4^+^), and **(E)** cytotoxic T type 17 (Tc17; CD8^+^IL-17^+^). Gating strategies are described in **Supplementary Figure 2**. Data from 3 independent experiments, with 3–4 mice per group. Bars represent mean ± standard error, analyzed by analysis of variance (ANOVA). *p < 0.05, **p < 0.01, ***p < 0.001, and ****p < 0.0001.

For CD8^+^ T lymphocytes analysis, we observed a significantly increased frequency of total CD8^+^ and activated CD8^+^CD69^+^ T cells in the lungs of mice treated against PD-1 in combination with AmB or monotherapy, in comparison to other treatments (**Figures 7A, B**). Notably, only the combined therapy resulted in a significant increase in the absolute numbers of these populations. We next evaluated cytokine-producing cytotoxic T lymphocyte subsets, Tc1 (IFN-γ^+^), Tc2 (IL-4^+^), and Tc17 (IL-17^+^), in the lungs of infected mice (**Figures 7C–E**). A consistent pattern emerged: anti-PD-1 administration, combined with AmB or alone, significantly elevated the frequencies of Tc1, Tc2, and Tc17 cells compared to both PBS and amphotericin B monotherapy. Again, the enhancement in absolute cell numbers was exclusive to the combination treatment, which demonstrated significantly higher counts of Tc1 and Tc17 cells compared to other groups, and a higher number of Tc2 cells compared to amphotericin B treatment.

## Discussion

Persistent antigenic stimulation during chronic infections can lead to a state of immune exhaustion, a phenomenon analogous to that observed in malignancies. This similarity has prompted the investigation of immune checkpoint inhibitors as a therapeutic strategy for severe chronic infections. PD-1/PD-L1 blockade has been shown to reinvigorate exhausted T cells and improve pathogen control in models of chronic viral (Barber et al., 2006; Ravi et al., 2014; Sangro et al., 2013), bacterial (Suarez et al., 2019; Zumla et al., 2016), and fungal (Lázár-Molnár et al., 2008; Wurster et al., 2022; Preite et al., 2024a) infections. Our group previously demonstrated that chronic PCM is characterized by significant upregulation of PD-1 on pulmonary CD4+ and CD8+ T cells, and the anti-PD-1 treatment decreased the expression of costimulatory molecules (ICOS, OX40L, and GITR) and coinhibitory molecules (PD-1 and PD-L1) induced by *P. brasiliensis* infection (Preite et al., 2024a). However, the efficacy of both immune checkpoint inhibitor monotherapy and conventional antimicrobial treatment in chronic infections can be constrained by microbial evasion mechanisms and risks of exacerbated immune response (Serris et al., 2022; Butler et al., 2011; Hafalla et al., 2012). To address these limitations, we sought to evaluate a novel combination regimen of anti-PD-1 and amphotericin B in *P. brasiliensis*-infected mice.

Prior to assessing combination effects, initial analysis confirmed the therapeutic efficacy of both monotherapies. Individual administration of anti-PD-1 or amphotericin B alone induced a significant reduction in pulmonary fungal burden after eight weeks of infection compared to PBS-treated controls. This is particularly relevant in PCM, where the lungs are the primary and most severely affected organ. The results obtained with AmB monotherapy, are align with its established fungistatic activity and historical role as first-line therapy for severe PCM (LeMonte et al., 2000; Peçanha et al., 2016). Notably, the combined therapy of anti-PD-1 and AmB demonstrated superior efficacy, promoting a further, significant decrease in the accumulated pulmonary fungal load that surpassed the outcomes achieved with either agent as a monotherapy. These results indicate that immune response modulation mediated by PD-1 blockade and the direct antifungal activity of AmB operate through complementary mechanisms, with the combination achieving substantially larger effect sizes than either monotherapy alone in controlling the infection.

The synergistic effect of the combination therapy was even more pronounced upon evaluation of secondary infection sites. PCM, while pulmonary-centric, is a disseminated disease; the liver and spleen constitute secondary target organs whose involvement, though typically less severe than in the lungs, is directly correlated with pulmonary disease severity and systemic dissemination. In the livers of animals receiving the anti-PD-1/AmB combination, we observed an approximately 50% greater reduction in fungal burden compared to either monotherapy group. A more dramatic effect was evident in the spleen, where a statistically significant reduction in fungal load was achieved exclusively with the combined treatment regimen. The synergistic effect was also reflected in pulmonary tissue preservation. Histopathological analysis revealed that lungs from animals receiving combined therapy contained few, well-delimited, and organized granulomatous lesions without evidence of rupture or pathogen escape, contrasting with the pathology observed in monotherapy groups. Morphometric analysis confirmed that the combined treatment resulted in the smallest area of compromised pulmonary tissue, surpassing the benefits of individual treatments. Given the improved pulmonary control and the direct systemic effects of the combination therapy on the liver and spleen, the therapeutic benefits observed at the tissue level translated directly into a significant survival advantage. While both the antifungal and the PD-1 blocker monotherapies significantly increased the survival of infected mice compared to controls, the combined treatment regimen demonstrated superior efficacy. Ultimately, mice groups that received monotherapies reached complete mortality at 150 days post-infection, whereas animals treated with the anti-PD-1/AmB combination achieved an 80% long-term survival rate. This marked improvement in overall survival is likely a direct consequence of the drastic reduction in fungal burden and the superior preservation of pulmonary architecture afforded by the synergistic action of the combined therapy.

Given the significant improvement after eight weeks of infection, we hypothesized that the disease in these animals was regressing. However, when a three-week treatment-free period was introduced post-therapy, the combination could not sustain disease regression, suggesting the protective effect wanes after treatment cessation. Therefore, we propose a new, prolonged treatment protocol consisting of two two-week therapy cycles separated by a one-week interval. This approach is supported by our previous work in PCM, where a similar prolonged scheme was necessary to sustainably modulate the immunosuppressive microenvironment and achieve lasting disease control (Preite et al., 2024b). With the implementation of this prolonged protocol, animals treated with the AmB and anti-PD-1 combination achieved the lowest fungal burden levels, which were significantly reduced compared to those receiving extended monotherapy with either agent alone. This superior microbiological clearance directly translated into a significant survival advantage. In subsequent mortality studies, mice receiving the prolonged combination therapy exhibited the longest survival times, significantly outperforming cohorts treated with prolonged AmB or anti-PD-1 monotherapy. This finding aligns with established therapeutic principles in other complex diseases, where optimized duration is critical for the success of combination regimens (Nunn et al., 2019; Gillis et al., 2021).

To understand the immunological basis for this superior outcome, we performed a detailed analysis of the immune response. Cytokine analysis in the lungs and liver of animals treated with the combination showed a general reduction in the levels of most quantified cytokines. While this might suggest a less vigorous immune response, it must be interpreted alongside the superior disease control and survival in this group. We propose that this cytokine reduction primarily reflects the more effective infection control, leading to diminished antigenic stimulation and a consequently lower demand for a sustained inflammatory response, a recognized physiological outcome following successful antimicrobial therapy (Serhan and Levy, 2018). This interpretation is strengthened by the profile of the anti-PD-1 monotherapy group, which showed elevated levels of IL-6, IL-2, and TNF-α, alongside reduced IL-4. This specific triad of cytokines is a well-established correlate with PD-1/PD-L1 axis blockade, signifying the reversal of T-cell exhaustion and the restoration of a robust Th1/Tc1 effector response. In cancer models, anti-PD-1 therapy is directly linked to increased production of pro-inflammatory and T-cell activating cytokines (Postow et al., 2018; Kamphorst et al., 2017; Howard et al., 2021). In the context of fungal immunity, this profile is indicative of a modulated and potentially more protective antifungal response (Galdino et al., 2018; Preite et al., 2023, 2024b). Thus, while the anti-PD-1 protective effect is mediated by immune activation, the combination with AmB achieves such effective pathogen control that it leads to a regression of the immune response compared with other treatments. The combination therapy significantly reduced immunosuppressive (IL-10, TGF-β) and Th2-associated (IL-4) cytokines, while preserving protective Th1/Th17 responses. This generalized cytokine attenuation, particularly in the lungs and liver, likely reflects more effective infection control, supported by reduced fungal burden, improved histopathology, and enhanced survival. These findings align with established roles of Th1/Th17 immunity in PCM resistance (Kashino et al., 2000; Tristão et al., 2017) and the association of IL-10, TGF-β, and IL-4 with severe disease (Ferreira et al., 2010; Costa et al., 2013).

Analysis of pulmonary infiltrates provided further corroboration for the efficacy of the combination treatment. Despite a reduction in overall cytokine levels, the lungs of mice treated with the anti-PD-1 and AmB combination exhibited a higher relative frequency of total leukocytes and myeloid cells. Notably, the increase in macrophage frequency following AmB treatment was somewhat unexpected. However, previous studies have shown that AmB can induce a protective Th1-type immune response (Cenci et al., 1997). Similar findings were reported in BALB/c mice infected with *A. fumigatus* spores and treated with AmB (Saxena et al., 1999). Furthermore, AmB has been shown to upregulate the pro-inflammatory cytokines IL-1β and TNF-α in mouse kidneys (Falk et al., 2005). The hypothesis that AmB exerts part of its therapeutic effect through immunomodulation is strengthened by the observation that its protective efficacy is compromised when mice are treated with neutralizing antibodies against TNF-α (Louie et al., 1995). In fungal model, blockade of PD-1/PD-L1 has been shown to reverse sepsis-induced immunosuppression by specifically enhancing MHC II expression on splenic antigen-presenting cells, including dendritic cells and macrophages (Chang et al., 2013). This aligns with our findings that activated macrophage frequencies (MHC-II^+^/CD86^+^) and activated dendritic cells (MHC-II^+^/CD86^+^) reached their maximum only in the combination treatment group. This demonstrates that the combination not only expanded these populations but also significantly augmented their activation state. The superior expansion and activation of these innate immune cells likely created a potent pro-inflammatory profile, which explains enhanced fungal clearance. This interpretation is strengthened by findings from a pulmonary aspergillosis model, where anti-PD-1 monotherapy improved survival, reduced fungal burden, and was associated with enhanced pulmonary leukocyte accumulation and pro-inflammatory cytokine production (Wurster et al., 2020). While we observed a reduction in overall lung cytokines in our combination therapy model, potentially due to more rapid antigen clearance and feedback regulation, the studies are concordant on the central role of PD-1 blockade in mobilizing and activating the pulmonary immune response. Therefore, the synergy observed in our study likely results from the AmB direct fungicidal and immunostimulatory effects converging with anti-PD-1 capacity to prevent the functional exhaustion of subsequently recruited antigen-presenting cells, a mechanism that directly translates to survival benefits, as also demonstrated by the superior outcomes of combination anti-PD-1 and caspofungin therapy (Wurster et al., 2020).

Analysis of lymphoid populations demonstrated that anti–PD-1 monotherapy altered CD4^+^ T-cell polarization without significantly affecting the total frequency of CD4^+^ T cells or activated. Specifically, anti-PD-1 led to a higher frequency of Th1 and Th17 cells, concomitant with a reduction in Th2 cell number. This pattern is consistent with data from Dulos et al., who reported that PD-1 blockade in human mononuclear cells increases antigen-induced CD4 responses toward Th1 and Th17 phenotypes, with a concomitant decrease in Th2 cytokines (Dulos et al., 2012). The addition of amphotericin B to anti-PD-1 treatment preserved the Th1 and Th17 higher frequencies, while the further decline in Th2 cells observed with PD-1 blockade alone was prevented. This may reflect AmB-mediated modulation, which could provide different polarizing signals than PD-1 blockade alone. Concomitantly, anti–PD-1 monotherapy induced a broad expansion of CD8^+^ T-cell subsets, increasing total CD8^+^ frequencies as well as activated, Tc1, Tc2, and Tc17 subsets. The addition of AmB did not alter the relative frequencies of these subsets compared to anti–PD-1 alone but yielded the greatest number of total CD8^+^ T cells, translating into the highest counts of Tc1 and Tc17 cells in the combination group. Mechanistically, this observation is supported by evidence that PD-1 signaling specifically suppresses the differentiation and expansion of CD8^+^ T cells, while PD-1 blockade enhances Tc1/Tc17 generation and plasticity, contributing to improved lung disease. Together, these data support a model in which anti–PD-1 therapy exerts potent effects on T-cell differentiation and expansion, shifting both CD4^+^ and CD8^+^ toward Th1/Th17 and Tc1/Tc17 phenotypes. When combined with AmB, this immune reprogramming is amplified in absolute cell numbers, potentially due to increased macrophage presence and survival, thereby expanding the pool of effector T cells capable of responding to fungal antigens.

In conclusion, our data show that combining PD-1 checkpoint inhibition with amphotericin B is a highly effective therapy for PCM, achieving huge effect sizes in fungal reduction, superior tissue preservation, and improved survival. This regimen achieves superior fungal clearance, tissue preservation, and survival compared with monotherapy, with efficacy dependent on an optimized, prolonged dosing schedule. The synergy likely arises from complementary mechanisms: amphotericin B exerts direct fungicidal and immunostimulatory effects that activate macrophages, while anti-PD-1 prevents T cell exhaustion. Together, they reshape the immunosuppressive environment of chronic PCM, inducing a robust Th1/Th17 and Tc1/Tc17 response essential for pathogen control. Notably, the reduced pulmonary cytokine levels in the combination group reflect resolution of inflammation following effective pathogen clearance. These findings support the broader application of immunotherapy-adjuvant synergy in systemic mycoses, offering a promising strategy for severe, chronic fungal infections.

## Data Availability

The original contributions presented in the study are included in the article/**Supplementary Material**. Further inquiries can be directed to the corresponding author.
