# Supplementary material for: Combined anti-PD-1 and amphotericin B therapy reduces fungal burden and enhances control of murine paracoccidioidomycosis
Source: Front Cell Infect Microbiol. 2026 Apr 2;16:1769296. doi: 10.3389/fcimb.2026.1769296 (PMC13083198; doi:10.3389/fcimb.2026.1769296)
Supplement: Supplementary file 1 [file Presentation1.pptx]

## Slide 1
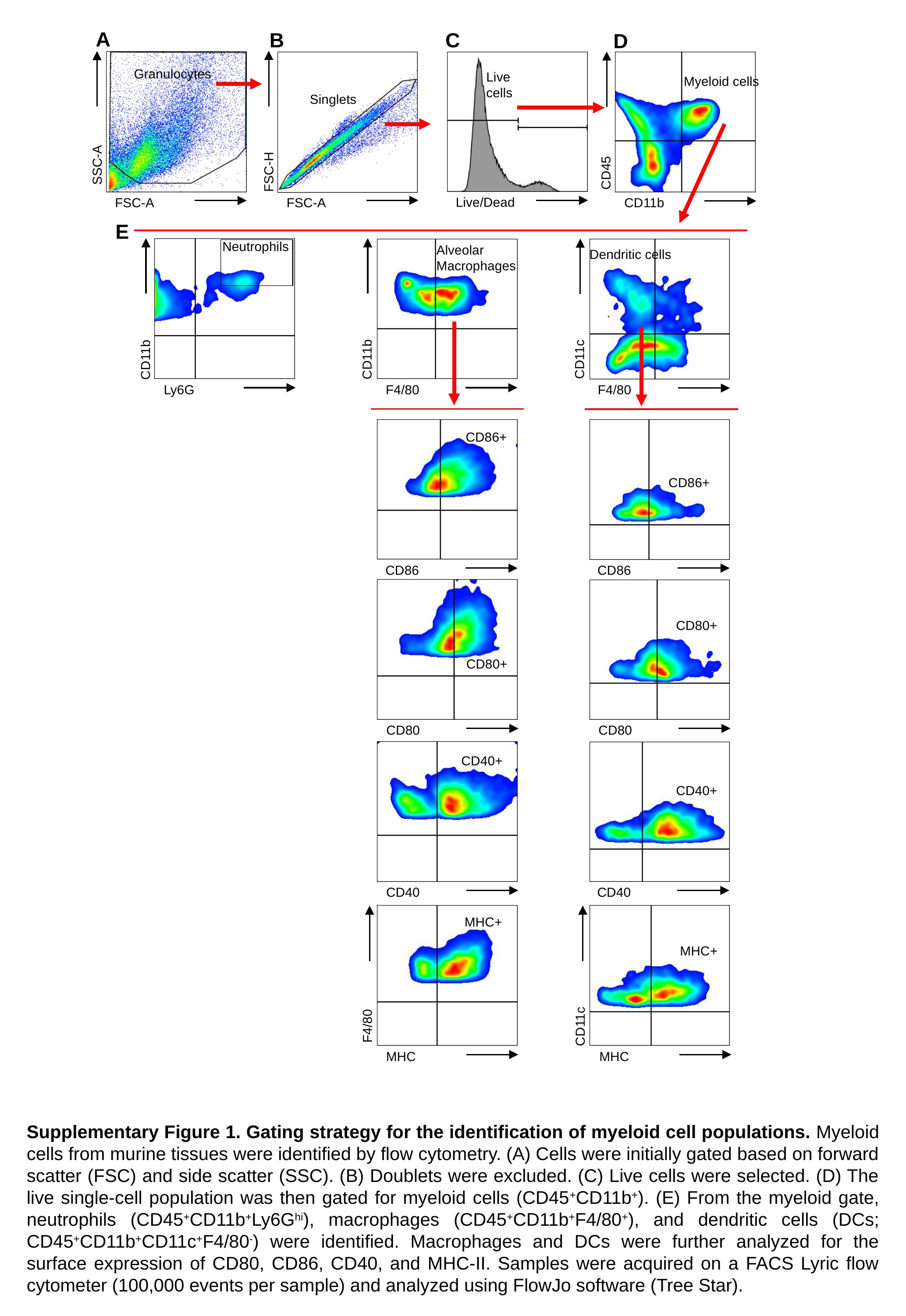

A
C
B
D
Granulocytes
Live cells
Myeloid cells
Singlets
SSC-A
CD45
FSC-H
Live/Dead
CD11b
FSC-A
FSC-A
E
Neutrophils
Alveolar
Macrophages
Dendritic cells
CD11c
CD11b
CD11b
F4/80
Ly6G
F4/80
CD86+
CD86+
CD86
CD86
CD80+
CD80+
CD80
CD80
CD40+
CD40+
CD40
CD40
MHC+
MHC+
F4/80
CD11c
MHC
MHC
Supplementary Figure 1. Gating strategy for the identification of myeloid cell populations. Myeloid cells from murine tissues were identified by flow cytometry. (A) Cells were initially gated based on forward scatter (FSC) and side scatter (SSC). (B) Doublets were excluded. (C) Live cells were selected. (D) The live single-cell population was then gated for myeloid cells (CD45+CD11b+). (E) From the myeloid gate, neutrophils (CD45+CD11b+Ly6Ghi), macrophages (CD45+CD11b+F4/80+), and dendritic cells (DCs; CD45+CD11b+CD11c+F4/80-) were identified. Macrophages and DCs were further analyzed for the surface expression of CD80, CD86, CD40, and MHC-II. Samples were acquired on a FACS Lyric flow cytometer (100,000 events per sample) and analyzed using FlowJo software (Tree Star).

## Slide 2
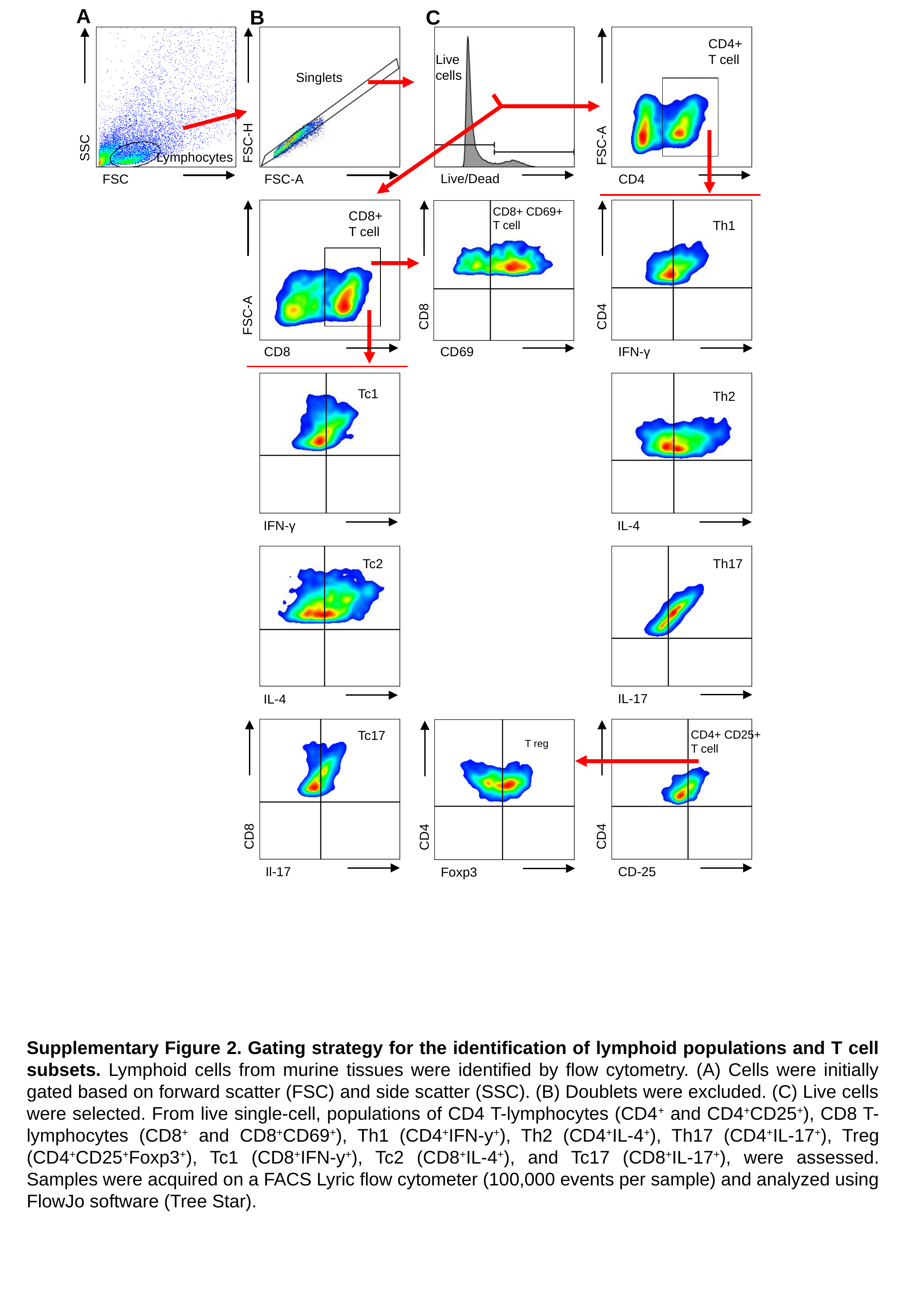

A
C
B
CD4+
T cell
Live cells
Singlets
FSC-H
FSC-A
SSC
Lymphocytes
Live/Dead
CD4
FSC-A
FSC
CD8+ CD69+
T cell
CD8+
T cell
Th1
FSC-A
CD8
CD4
CD8
CD69
IFN-γ
Tc1
Th2
IFN-γ
IL-4
Tc2
Th17
IL-17
IL-4
Tc17
CD4+ CD25+
T cell
T reg
CD8
CD4
CD4
Il-17
CD-25
Foxp3
Supplementary Figure 2. Gating strategy for the identification of lymphoid populations and T cell subsets. Lymphoid cells from murine tissues were identified by flow cytometry. (A) Cells were initially gated based on forward scatter (FSC) and side scatter (SSC). (B) Doublets were excluded. (C) Live cells were selected. From live single-cell, populations of CD4 T-lymphocytes (CD4+ and CD4+CD25+), CD8 T-lymphocytes (CD8+ and CD8+CD69+), Th1 (CD4+IFN-y+), Th2 (CD4+IL-4+), Th17 (CD4+IL-17+), Treg (CD4+CD25+Foxp3+), Tc1 (CD8+IFN-y+), Tc2 (CD8+IL-4+), and Tc17 (CD8+IL-17+), were assessed. Samples were acquired on a FACS Lyric flow cytometer (100,000 events per sample) and analyzed using FlowJo software (Tree Star).

## Slide 3
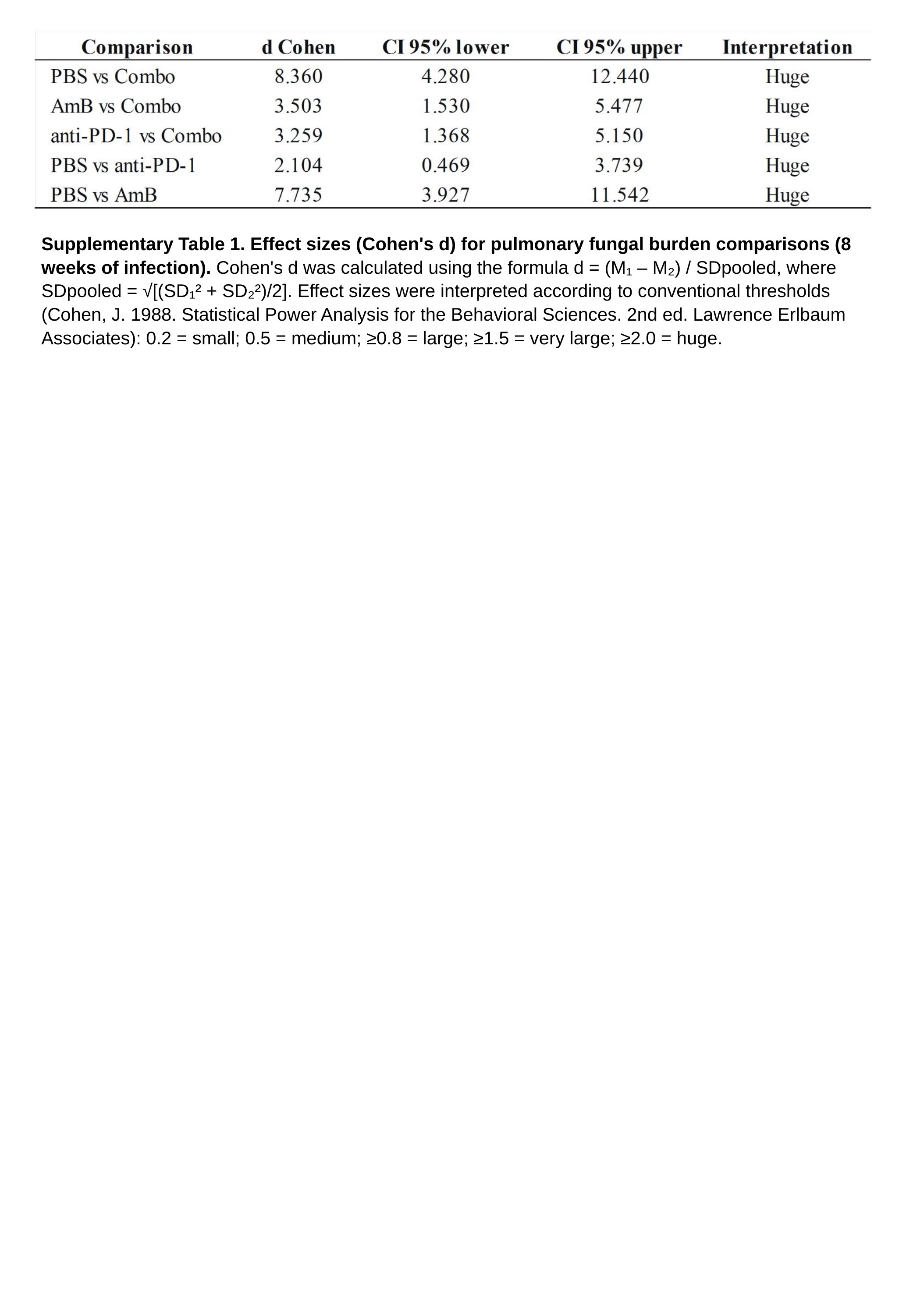

Supplementary Table 1. Effect sizes (Cohen's d) for pulmonary fungal burden comparisons (8 weeks of infection). Cohen's d was calculated using the formula d = (M₁ – M₂) / SDpooled, where SDpooled = √[(SD₁² + SD₂²)/2]. Effect sizes were interpreted according to conventional thresholds (Cohen, J. 1988. Statistical Power Analysis for the Behavioral Sciences. 2nd ed. Lawrence Erlbaum Associates): 0.2 = small; 0.5 = medium; ≥0.8 = large; ≥1.5 = very large; ≥2.0 = huge.
